# Identification of Flying Insects in the Spatial, Spectral, and Time Domains with Focus on Mosquito Imaging

**DOI:** 10.3390/s21103329

**Published:** 2021-05-11

**Authors:** Yuting Sun, Yueyu Lin, Guangyu Zhao, Sune Svanberg

**Affiliations:** 1 Guangdong Provincial Key Laboratory of Optical Information Materials and Technology & Center for Optical and Electromagnetic Research, South China Academy of Advanced Optoelectronics, South China Normal University, Guangzhou 510006, China; yuting.sun@coer-scnu.org (Y.S.); yueyu.lin@coer-scnu.org (Y.L.); Guangyu525@gmail.com (G.Z.); 2 National Center for International Research on Green Optoelectronics, South China Normal University, Guangzhou 510006, China; 3 Department of Physics, Lund University, P.O. Box 118, SE-221 00 Lund, Sweden

**Keywords:** insects, mosquito, spectroscopy, wing-beat frequency, image

## Abstract

Insects constitute a very important part of the global ecosystem and include pollinators, disease vectors, and agricultural pests, all with pivotal influence on society. Monitoring and control of such insects has high priority, and automatic systems are highly desirable. While capture and analysis by biologists constitute the gold standard in insect identification, optical and laser techniques have the potential for high-speed detection and automatic identification based on shape, spectroscopic properties such as reflectance and fluorescence, as well as wing-beat frequency analysis. The present paper discusses these approaches, and in particular presents a novel method for automatic identification of mosquitos based on image analysis, as the insects enter a trap based on a combination of chemical and suction attraction. Details of the analysis procedure are presented, and selectivity is discussed. An accuracy of 93% is achieved by our proposed method from a data set containing 122 insect images (mosquitoes and bees). As a powerful and cost-effective method, we finally propose the combination of imaging and wing-beat frequency analysis in an integrated instrument.

## 1. Introduction

Insects exhibit the largest variety of species in the animal kingdom with an estimated number of 5.5 million varieties [1], and represented by 1 gigaton of carbon, they account for the largest fraction of the total animal biomass [2]. Clearly, they play a very important part in the global ecosystem, and like the rest of the biosphere, are also influenced by global change [3]. Among the numerous types of insects, pollinators, disease vectors and agricultural pests all have a pivotal influence on society. Pollinators are indispensable in food production, and in the absence of insects the diet would be very limited and meager [3]. Disease vectors include malaria-carrying mosquitos, such as *Anopheles*, which transfer *Plasmodium* parasites, responsible for about 400,000 deaths annually, mostly children in Africa [4]. Additional vectors, including for dengue fever, West Nile and Zika virus, Japanese encephalitis, and yellow fever, account for a further 300,000 deaths annually [5]. Agricultural pests, such as classical locusts, army worms, plant hoppers, etc. are responsible for strong crop losses in many parts of the world. Monitoring and control of such insects have a high priority, and automatic systems are highly desirable. Many of the insects of interest have wings and can move over considerable distances. While capture and analysis by biologists constitute the gold standard in insect identification, optical and laser techniques have the potential for high-speed detection and automatic identification when insects are in flight. Analysis can be based on shape, spectroscopic properties such as reflectance, depolarization, and fluorescence, as well as wing-beat frequency monitoring. Many approaches can be implemented in remote-sensing systems such as time-of-flight- (TOF), or CW bi-static light detection and ranging (lidar) installations, while additionally, imaging followed by processing could be available in in situ insect traps.

The present paper discusses these approaches, and in particular presents a novel method for automatic identification of mosquitos based on image analysis. Details of the procedure are presented, and selectivity is discussed. Before going into our method for insect identification by image processing, we will briefly describe techniques based on spectroscopy and wing-beat frequency analysis. We will especially relate to our own experience, and in a concluding section discuss pros and cons for different approaches. Finally, we propose a cost-effective system based on basic image processing and wing-beat frequency analysis implemented in an insect trap.

### 1.1. Reflectance, Depolarization, and Fluorescence Spectroscopy

Spectroscopic approaches (see, e.g., [6,7]) interrogate the molecular constituents of objects, naturally then also including insects. Photonic interactions related to atmospheric as well as aquatic fauna were recently reviewed in [8]. The reflectance spectrum recorded following illumination with a broad spectral distribution of radiation corresponds to the perceived color, but of course also extends outside the region accessible to the human eye, i.e., to the ultraviolet (UV) and infrared (IR) domains. Surface reflections (specular reflection) do not carry information on the interior of the material, which the diffuse light does. Then, scattering inside the substance occurs, and specific absorption, governed by the Beer-Lambert law, is revealed as color in a generalized sense. Specifically, polarization analysis can be performed, and the depolarization of primary impinging linearly polarized light can carry information on the structure of an insect in terms of hairiness, microstructures, etc. In particular, insects can be distinguished from raindrops [9], which according to Mie scattering theory exhibit no depolarization. We note that colors also can result from ordered nanostructures in, e.g., insect wings and bodies. Such structural colors, which are among the most spectacular in the animal kingdom, are due to interference effects [10].

Reflection spectra from flying insects were first reported in [11], where a simple amateur astronomical telescope, connected to a compact digital spectrometer, was directed horizontally towards a background termination, arranged to be as dark as possible and placed at a distance of about 100 m. When white sunlight reflected off insects flying into the telescope field-of-view, abrupt spikes rising up from the low background were spectrally recorded. Species of damselflies were identified. Similar dark-field work employing sunlight, but also an artificial lamp at night time, was reported in [12] for the case of Chinese agricultural pests. Laboratory-controlled studies on released insects gave detailed information on the reflectance spectra of certain pests, and corresponding fluorescence spectra were also recorded [13]. Later, full multi-spectral reflection imaging of insects using an imaging spectrometer was also accomplished; see, e.g., [14].

Fluorescence, induced by a UV laser or other radiation source, and appearing Stokes-shifted towards longer wavelengths, can carry complementary information on the molecular constituents, although spectral structures are, as for reflectance, broad with overlapping contributions from major constituents such as melanin, carotenoids, etc., pertinent to insects. Sharper Raman structures due to characteristic molecular vibrations may also be observed for major constituents. Fluorescence spectra from insects were reported in [15,16] using pulsed-laser TOF lidar techniques, which have a wide applicability in remote-sensing environmental monitoring and the study of cultural heritage (see, e.g., [17,18,19]). By first dusting caught and identified insects with fluorescent dyes, unique spectral signatures are obtained, and the dispersion of insects in their natural habitat can be studied [16]. Fluorescence lidar monitoring of Chinese agricultural pests were also performed [20] using TOF lidar techniques, and could also be extended to flying birds [21].

### 1.2. Wing-Beat Frequency Analysis

Flying insects flap their wings with frequencies, which are related to the species, and also to the sex within the same species. This gives rise to well-known sounds with typical pitch, which are readily recognized by humans as being different for mosquitos, flies, and bees. Clearly, analyzing the sounds captured by a microphone adds specificity to the approach. Thus, acoustic systems employing pressure waves for insect identification have been developed (see, e.g., [22,23]). Wing-beats can also be detected as oscillation in the light, which scatters off a flying insect. This can be observed in passive, dark-field, systems using ambient light [12], but more conveniently using high-repetition-rate or CW lidar systems. First results were related to lidar monitoring of bees, as reported in [24,25], and with the application to the detection of hidden land-mines and explosives when using honey bees, which were specially trained to be attracted to TNT fumes. Insect detection and associated wing-beat frequency analysis at considerable range were made highly realistic with the introduction of CW, bi-static lidar systems based on the Scheimpflug principle [26]. Such systems, which are also very powerful for aerosol and air pollutant monitoring, are described, e.g., in [27,28,29]. Scheimpflug lidars observe a transmitted CW laser beam at an angle with a low-price amateur astronomy telescope, placed less than a meter from the transmitting optics and on a co-rotated platform. The imaging detector is arranged at a specific angle, for which the laser beam is imaged sharply on the detector array, at close as well as at far range. In contrast to TOF lidar systems, where the signal intensity falls off with an 1/r^2^ dependence (r being the range), a Scheimpflug lidar basically shows a constant signal level out to large ranges, but now with a strongly falling-off range resolution. Since the array detector can be read out at high speed, a high temporal resolution is obtained, also allowing wing-beat frequencies up to hundreds of Hz to be recorded, and with a capability of one hundred thousand observed events for single-night recordings.

Such systems have been employed in many field experiments on different continents, where monitoring of wing-beat frequencies was also performed (see, e.g., [30,31,32,33,34]. Analysis of light depolarization [9], insect flight speed [33], and differential back-scattering using two laser wavelengths (see, e.g., [35,36,37]) has also been accomplished. Normally, Scheimpflug lidar systems operate with elastic back-scattering from the targets. However, by using a double Scheimpflug arrangement, with a 2-D detector, the fluorescence spectrum induced by a blue CW laser can be recorded for each range interval. This was demonstrated for under-water lidar monitoring at ranges up to 5 m [38,39], and also for terrestrial vegetation monitoring from a light-weight drone-based system [40].

It should be noted that several non-lidar laser-based systems have been constructed for in situ or close-range characterization of insects [41,42,43,44]. These include systems with different laser or LED wavelengths, allowing crude reflectance characterization, as well as studies of depolarization. Below, we will concentrate on in situ sampling systems, which combined with optical detection have the potential for be particularly realistic and cost-effective.

## 2. Mosquito Sampling Method

### 2.1. Common Sampling Methods

We will here first describe current mosquito sampling methods and then present the system used in our imaging study. The main conventional methods for monitoring adult mosquitoes are the human-landing catch, human-baited double net trap, and light traps techniques. The human-landing catch approach uses a tube for mosquitoes trying to penetrate the human skin [45]. This method puts collectors at risk of being bitten by infectious mosquitoes and contracting malaria, which is of course very problematic. The human-baited double net trap is divided into an inner net and an outer net [46]. The attractant sits in the enclosed area of an inner net, exposing free skin areas; the collector then uses an electric mosquito sucker between the inner net and the outer net to collect mosquitoes, which settled on the mosquito net. Since the distance between the attractor and the mosquitoes is larger, the mosquito trapping effect may be reduced accordingly. The light trap method utilizes the phototaxis of mosquitoes. The mosquito light trap is often placed near mosquito breeding grounds [47]. It has limitations such as easy damage to the equipment, and attraction of also other types of insects subject to phototaxis.

### 2.2. Present System Description

**Figure 1:**
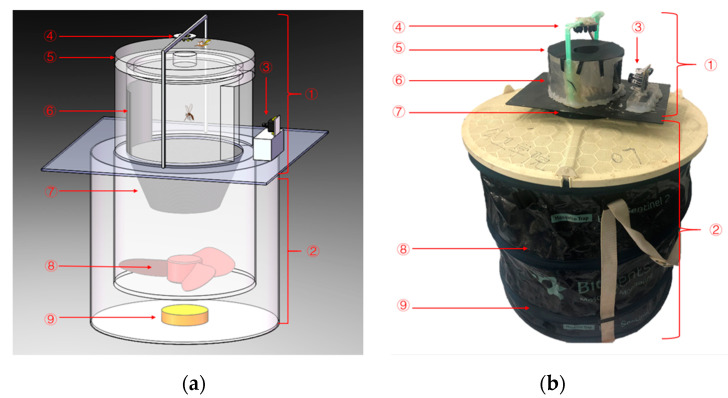
Overall structure of the instrument, consisting of an image recording section ① placed on top of a BG-Sentinel commercial mosquito trap ②. ③ indicates a camera for taking pictures and ④ is an illuminator placed on the top of the instrument. The cover has a small opening ⑤ on top. Black cloth ⑥ is used as the scene background. Mosquitoes are caught in a black net bag ⑦. A fan ⑧ is placed under the mosquito net bag, and at the bottom of the container there is an attractant ⑨. (**a**) shows the design structure of the system, while 1 (**b**) is a physical photo of the system.

**Figure 2:**
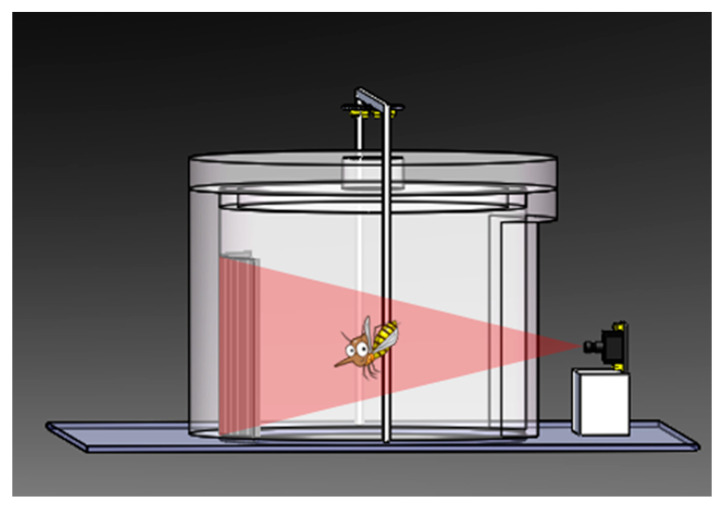
Upper part of the instrument with camera observing insects, which are illuminated from above, against a black cloth background.

The instrument designed for the present experiments avoids certain problems of the types mentioned above. The structure of the arrangement is shown in Figure 1 and Figure 2. We use a Biogents BG-Sentinel commercial trap [48], on top of which we install the imaging unit. The two parts are marked with ① and ② in Figure 1. An illuminator ④ is placed on the top of the instrument. On the one hand, it fulfills the function of light trapping, and on the other hand, it provides illumination for the photos taken by the camera ③. On the opposite side of the camera, the instrument uses black cloth ⑥ as the scene background, which increases the contrast in the photos of the insects taken by the camera.

The lower BG-Sentinel trap part of the instrument is a foldable white fabric cylindrical container, 40 cm high and 36 cm in diameter. At the bottom of the container is an attractant ⑨ that simulates human odor. The middle part of the instrument is connected to a black mosquito catching net bag ⑦. A fan ⑧ is placed under the mosquito net bag, and causes an air flow to suck mosquitoes into the mosquito net bag. The air flow effectively prevents the trapped mosquitoes from flying again and the bag ensures that the mosquitoes will not be damaged by the fan.

The light and the odor attractant bring mosquitoes to the instrument, where they enter a small opening ⑤ on the top, as caught by the air flow produced by the fan. The camera will take pictures of the mosquitoes sucked into the instrument. The photos are transferred to a personal computer, where insects are counted and classified as described in Section 3.2. Our image recognition algorithm effectively distinguishes mosquitoes from some other phototaxis insects captured by the instrument, ensuring a high accuracy of the data. The cost of our instruments is very low, and it is quite sturdy and can be deployed for large-scale, long-term monitoring of mosquitoes. It can save a lot of manpower and material resources.

In our case, the sampling experiments were performed in the laboratory environment. Mosquitoes and our system were put in the same mosquito net enclosure to make sure that mosquitoes could fly freely into our system and they could not move outside the laboratory. The camera we used is an ELP-USB130W01MT-L170 unit. The sampling platform is Labview 2014 (32 bit) based on Windows 7, 16 G memory and Intel (R) Core (TM) i7-3770 3.40 GHz.

## 3. Insect Imaging

Many advanced methods have been proposed to detect and classify insects. A solution was presented in [49], to detect *Aedes Aegypti* mosquito species using images taken from a camera with a 500× optical zoom and employing a support vector machine (SVM) algorithm. By employing the SVM algorithm, properties like the mean value, standard deviation, entropy, energy, contrast, correlation, and eccentricity are considered. An accuracy of 80% was obtained from the processes. Fixed threshold and sliding threshold methods were introduced in [50] to detect insects by using smart phones. By improving the existing technique based on a fixed threshold method, an accuracy of 95% was obtained in online identifying and counting of insects. A system for combating infectious diseases by using image classification techniques and collaboration with ordinary citizens was introduced in [51]. Citizens were asked to use their smart phones for image capturing and reporting mosquitoes, which they encountered. This approach is capable of using computer vision techniques to strengthen communities affected by an arbovirus epidemic and provide valuable information to experts being in charge of coordinating solutions. A further application employing smart phones is presented in [52]. A system, which integrates image processing, feature selection, unsupervised clustering, and a support vector machine (SVM) learning algorithm for classification, was introduced in [53]. This system can with high accuracy classify nine different disease-carrying species, which were captured from a real outdoor trap. A data set containing 303 tagged images of nine mosquito species taken via a smart-phone camera was used in this paper. In the processing step, 39 features based on local binary patterns [54] and Haralick texture features [55] were extracted, and then the number of features was reduced to 8 through linear discriminant analysis (LDA), and finally fed to an SVM for classification. The overall accuracy of the system for nine species is 77.5%. A vision-based architecture of detection with You Only Look Once (YOLO) and classification with SVM algorithms was introduced in [56]. A data set with six species of flying insects comprised 700 individuals. Shape feature, texture feature, color feature, and HOG feature were extracted to train the SVM algorithms. The accuracy of counting and classification is above 90% and the miss rate is below 7.5%.

However, some of these methods need plenty of calculations and data to train the algorithms, which make them both time-consuming and expensive. These methods can be implemented in economically developed areas because these places have sufficient conditions of support, but most of the areas affected by mosquito-borne diseases are less developed and poor. The most important thing for the monitoring of mosquito populations should be convenience and reliability, so that it can be carried out easily under the complex conditions in these areas. Our purpose is to develop a fast, convenient, and effective method with low-cost that can simultaneously have the effects of population monitoring, classification and capturing.

We here present a new simple and effective method with low cost and high accuracy. The main idea of our method is template matching [57]. Template matching involves defining a measure or a “cost” to find the “similarity” between the (known) reference patterns and the (unknown) test patterns by performing a matching operation. Since template matching was originally proposed, many improved algorithms such as Fast Template Matching [58] and Very Fast Template Matching [59] were introduced with higher speed and better performance. Using a single template, the detection capacity is clearly very limited. Multi-target recognition is the most common case in object detection. Multi-target template matching algorithms were developed based on single template matching. Classification is also another common task in object detection. Without employing large data sets and performing excessive calculations, a simple way with high accuracy is introduced in the present paper.

### 3.1. Detection and Classification

In this section we present our method to detect and classify mosquitoes from other insects captured by the camera. The experimental environment of detection and classification processes is Visual Studio 2019 based on Windows 10, 16 G memory and Intel (R) Core (TM) i5-4210U 1.70 GHz. We chose bees as an example of a species, to be discriminated against. There is a sequence of steps in our method, including image graying, padding, template matching, covering, and classification, as detailed below.

**Figure 3:**
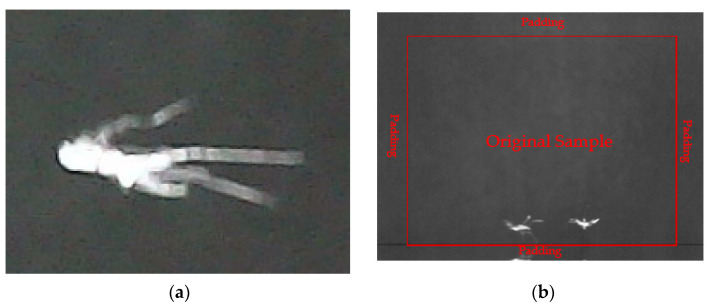
(**a**) Template image; (**b**) Image sample after padding.

- **Step one:** Convert the image (pixels: 1280 × 960) into grayscale.
- **Step two:** Cut out the template (pixels: 200 × 144) from one image sample, as shown in Figure 3a, and perform the padding operation to another image sample. Padding operation means to increase length and width of the original photo with the length and width of the template to make the new image (pixels: 1480 × 1104), as shown in Figure 3b. Without applying a padding operation, targets that are near the boundary would be missed by the algorithm.

First, align the template in the upper left corner of the new image. Calculate the similarity between the template and the area covered by the template (the Region of Interest, ROI). The correlation coefficient $r_{ccoeff}$ is calculated as follows [57]:

(1) $$r_{ccoeff}\left(x,\;y\right)=\sum_{x^{\prime },y^{\prime }}I_{T}^{\prime }\left(x^{\prime },y^{\prime }\right)\times I_{ROI}^{\prime }\left(x+x^{\prime },y+\;y^{\prime }\right)$$

Here, x and y are the pixel location information in the image; 0≤x<1280 and 0≤y<960. $x^{\prime }\;\mathrm{and}\;y^{\prime }$ are the pixel location information in template; $0\leq x^{\prime }<200$ and $0\leq y^{\prime }<144,\;$ We further calculate $I_{T\;}^{\prime }$ [57]:

(2) $$\;I_{T}^{\prime }\left(x^{\prime },y^{\prime }\right)=\frac{I_{T}\left(x^{\prime },y^{\prime }\right)-\frac{\sum_{x^{\prime \prime },y^{\prime \prime }}I_{T}\left(x^{\prime \prime },y^{\prime \prime }\right)}{W\times H}}{\sqrt{\sum_{x^{\prime \prime },y^{\prime \prime }}I_{T}^{2}\left(x^{\prime \prime },y^{\prime \prime }\right)}}$$

$I_{T}\left(x^{\prime },y^{\prime }\right)$ is the intensity of the pixel, which is located in $\left(x^{\prime },y^{\prime }\right)$ of the template. $x^{\prime \prime }\;\mathrm{and}\;y^{\prime \prime }$ are also the pixel location information in the template, $\;0\leq x^{\prime \prime }<200$ and $0\leq \;y^{\prime \prime }<144$. W and H are the width and height of the template, which are 200 and 144, respectively. Equation (2) can be considered as a normalization process. By subtracting the mean and dividing by the variance, it is guaranteed that the particular light intensity will not affect the calculation results.

Further, $I_{ROI}^{\prime }$ is calculated as [57]:

(3) $$I_{ROI}^{\prime }\left(x^{\prime },y^{\prime }\right)=\frac{I_{ROI}\left(x^{\prime },y^{\prime }\right)-\frac{\sum_{x^{\prime \prime },y^{\prime \prime }}I_{ROI}\left(x^{\prime \prime },y^{\prime \prime }\right)}{W\times H}}{\sqrt{\sum_{x^{\prime \prime },y^{\prime \prime }}I_{ROI}^{2}\left(x^{\prime \prime },y^{\prime \prime }\right)}}$$

Here $I_{ROI}\left(x^{\prime },y^{\prime }\right)$ is the intensity of the pixel, which locates in $\left(x^{\prime },y^{\prime }\right)$ of the ROI region. $x^{\prime \prime }$ and $y^{\prime \prime }$ are also the pixel location information in the ROI; $0\leq \;x^{\prime \prime }<200$ and $\;0\leq y^{\prime \prime }<144$. Again, W and H are the width and height of the template, which are 200 and 144, respectively.

**Figure 4:**
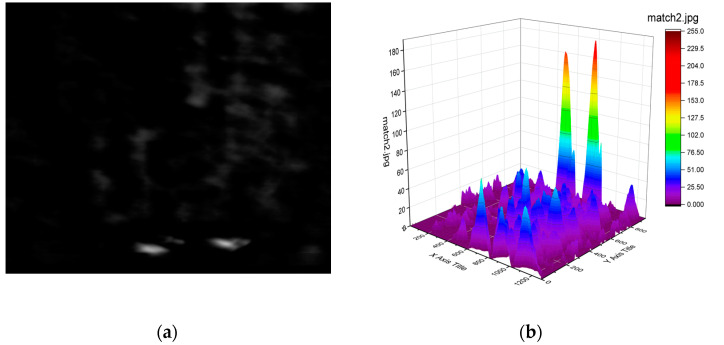
(**a**) New array $R_{ccoeff}$; (**b**) 3D display of $R_{ccoeff}$, where the vertical scale actually shows $R_{ccoeff}\;$ multiplied by 255, for better clarity.

- **Step three:** Move the template one pixel to the right and repeat the calculation in Step 2 until the template arrives to the far right.
- **Step four:** When the template arrives to the far right, move it one pixel down and repeat the calculation in Step two and Step three from the far left.
- **Step five:** After Step three and Step four, we can get a new matrix $R_{ccoeff\;}$ of dimension (1480 − 200 + 1) × (1104 − 144 + 1), composed of the calculated correlation coefficient *r_ccoeff_* values. They are limited between −1 and 1. The higher the correlation value is, the greater the matching degree is. The result is shown in Figure 4b), where the vertical scale, showing the correlation, has been multiplied by 255 for clarity. Then we select the maximum value, the minimum value and maximum position information from the matrix.

Three threshold values, 0.4, 0.45, and 0.5, were tested in the first place. When the threshold value is set too small, mosquitoes that do not match the template very well can be detected, which improves the detection efficiency, but at the same time some interference factors, such as debris from trees, feathers etc. may be mistakenly detected as mosquitoes, which reduces the detection accuracy. On the contrary, when the threshold value is set too high, some mosquitoes will be ignored by the algorithm because the matching coefficient is not high enough, which reduces the detection efficiency, but at the same time the algorithm will also eliminate some interfering factors to improve the detection accuracy. After testing and comparison, the algorithm is found to have the best combination of detection rate and accuracy when the threshold value of 0.4 is selected. When the calculated correlation coefficient is higher than the threshold value, we define that it belongs to the location area of the object.

- **Step six:** Centering on the coordinates of the maximum, an area of the same size (pixels: 200 × 144) as the template in the original image (pixels: 1280 × 960) is placed. Normalization, binarization, and morphological processing are carried out for the region within the original area to obtain the contour of the object. Then, we calculate the area and perimeter of this contour and divide the perimeter by the area to get the ratio.
- **Step seven:** In the matrix $R_{ccoeff}$ covering procedure is performed, which is replacing the area in Step six with the minimum value from Step five. Then, we repeat the process in Step five and Step six until the maximum value is smaller than the threshold.

### 3.2. Result Evaluation

#### 3.2.1. Detection Algorithm Evaluation

**Figure 5:**
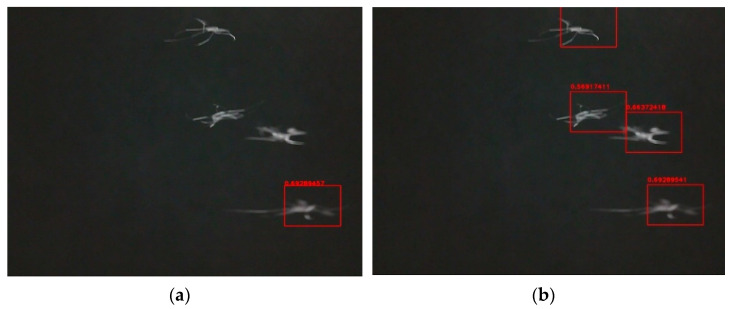
(**a**) Single template matching; (**b**) Multi-target template matching.

**Figure 6:**
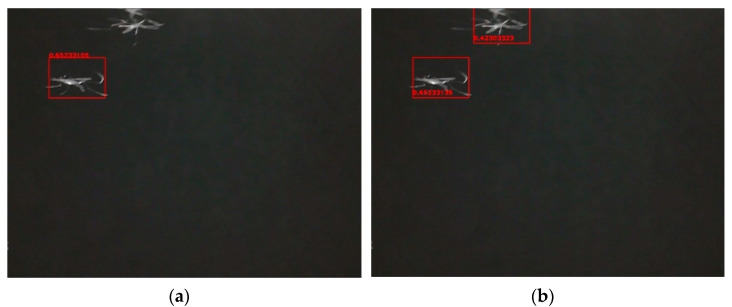
(**a**) Without padding; (**b**) With padding.

Single template matching is the main idea of our proposed method. On the basis of signal template matching, a multi-target template matching algorithm was developed by adding a covering procedure. In order to further improve the detection efficiency, a padding operation was introduced to multi-target template matching, which constitutes the detection algorithm we proposed. Fuzzy preprocessing operation was not considered in the steps. In fact, Fuzzy preprocessing can reduce imaging interference caused by various kinds of uncertainties. It has been shown that it is possible to further improve the recognition accuracy by performing a Fuzzy preprocessing operation on the image [60,61]. To evaluate the performance of these three types of detection algorithms and the degree of possible improvement, we recorded 122 insect images (71 images of mosquitoes and 51 images of bees) and examples of results from the three different algorithms are shown in Figure 5 and Figure 6. Figure 5 shows the different results between single template matching and multi-target template matching. After adding the covering procedure, more than one target can be detected. The target (mosquito) near the boundary cannot be found by our algorithm in Figure 6a. By padding the boundary, there are more pixels near the target that can be used when calculating (see Figure 6b). Table 1 shows the different detection rates for these three algorithms, which includes Single template matching, Multi-target template matching and our proposed detection algorithm. After our improvements, the detection rate reaches 92%, from an initial 64%.

#### 3.2.2. Classification Method Evaluation

**Figure 7:**
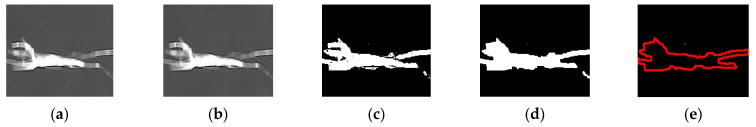
Processed sample of mosquito: (**a**) Original ROI; (**b**) Normalization; (**c**) Binarization; (**d**) Morphological processing; (**e**) Classification results.

**Figure 8:**
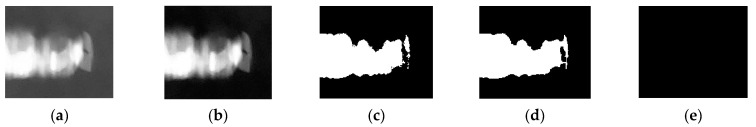
Processed sample of bee. (**a**) Original ROI; (**b**) Normalization; (**c**) Binarization; (**d**) Morphological processing; (**e**) Classification results.

**Figure 9:**
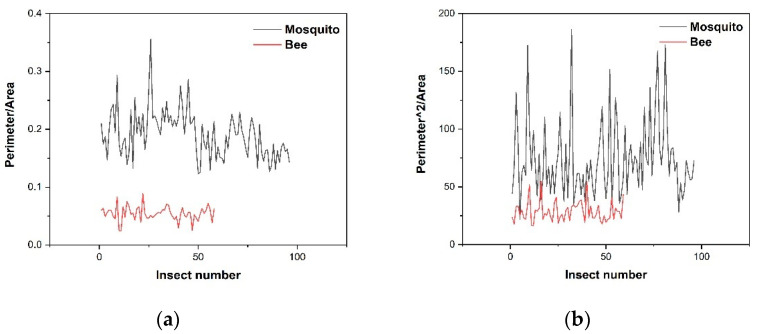
(**a**) The perimeter/area values for mosquitoes and bees. (**b**) The (perimeter)^2^/area values for mosquitoes and bees.

To evaluate the performance of the classification methods, several pictures of bees were also recorded by the camera. After Step five in Section 2.1, a set of processes were performed with the ROI to classify mosquitoes and bees. The processes are displayed in Figure 7 and Figure 8. We can see, from Figure 7d and Figure 8d, that the body area of a bee is much larger than that of a mosquito. At the end of the processing step, only those targets classified as mosquitoes by the algorithm will be colored, otherwise there will be no operation. Different results are shown as Figure 7e and Figure 8e. For crude classification we calculate the area and perimeter of the target in the ROI. Perimeter means the number of pixels contained in the edge of the contour, while area means the number of all the pixels contained in the contour. Using these parameters, we calculate two contrast functions, perimeter/area and (perimeter)^2^/area. The two function values for each mosquito and each bee are plotted in Figure 9a,b. We note that while the second function (plotted in (b)) is dimensionless [62], i.e., only depends on shape/structure, the first function (plotted in (a)) also depends on size. We note, that shape/structure (the main information from imaging) alone can discriminate between the insects, but when also incorporating size (a), the discrimination becomes better.

In order to objectively evaluate the effectiveness of the method, several evaluation criteria were introduced as shown in Equations (4)–(6), where *TP*, *FN*, and *FP* are defined as follows. *TP*: true positive, which means that objects were detected as mosquitoes and they are mosquitoes. *FN*: false negative, which means that objects are not detected as mosquitoes but they are mosquitoes. *FP*: true negative, which means that objects were detected as mosquitoes but are not mosquitoes (which could be absence of insects, or a bee). Then *recall*, *precision* and *F-measure* values, which are standard performance parameters, can be calculated as follows [63]:

(4) $$Recall=\frac{TP}{TP+FN}$$

(5) $$Precision=\frac{TP}{TP+FP}$$

(6) $$\;\mathrm{F-}measure=2\times \frac{\;Precision\times Recall}{Precision+Recall\;}$$

Sometimes there are contradictions between *recall* and *precision*. In this case, we need to carry out a synthesis. *F-measure* is defined as a harmonic mean of *recall* and *precision* [64]. The higher the *F-measure*, the better performance will be. We have earlier successfully used these performance criteria in connection with the extraction of important information from cluttered images [65]. The performance results are presented in Table 2. No Classification means that we only used the detection method, while Classification means that we used the detection method and the Classification method at the same time. Compared with the No Classification method, all the values of the classification methods including *recall*, *precision*, and *F-measure*, are increased. We note that there is a quite limited number of samples in this evaluation. More advanced and accurate detection methods and processing methods require a large number of samples to support, which may be implemented in the near future.

## 4. Conclusions and Future Work

In this paper, we have reviewed optical methods for insect characterization based on spectroscopic features such as reflectance, depolarization, and the use of wing-beat frequency analysis. Such techniques can be implemented in remote sensing systems based on TOF or compact CW bi-static lidar systems, but also in in situ insect traps employing, e.g., chemical attraction. CW systems, based on the Scheimpflug principle, are found to be particularly powerful in the continuous logging and characterization of huge amounts of insects, which intersect the laser beam at different distances. Traps have been extensively used for later detailed manual analysis of species, but clearly are very labor intensive. We have here presented a method for image analysis of insects entering a trap, which operates with combined light, suction, and chemical attraction. Images are recorded by a low-cost camera when illuminated insects are passing the field-of-view at a defined distance in the suction channel. In this way we ensure that the target apparent size as captured by the camera does not change greatly, which facilitates the analysis of the images stored in the computer. Template matching and simple shape features (perimeter and area) were used for the classification. After data processing, an accuracy of 93% was obtained in automatic discrimination between mosquitoes and bees. Compared with spectroscopy techniques, such image capture and analysis can be implemented very cost-effectively using compact systems.

Clearly, there is much room for improvement in the approach taken. Sharper images can be captured with a higher-quality camera, and matched to an extensive bank of template images. Machine learning or deep learning approaches could be implemented. In such a way, more detailed speciation would become feasible, which would be particularly valuable for differentiating different species of mosquitos, and even sexes. Needless to say, the processing then becomes correspondingly more demanding.

The generalization ability will be the important factor to consider when choosing machine learning or deep learning approaches. The generalization refers to the adaptability of machine learning algorithms to fresh samples. The actual application of a neural network depends on its generalization, meaning that the generalization ability absolutely determines whether the structure of a neural network is effective. There have been many solutions to the generalization of deep learning, such as various gradient descent methods, network structure improvements (including activation functions, connectivity styles), etc. A two-stage training method including pre-training processing and implicit regularization training processing was presented in [66]. Compared with existing methods, the two-stage method had better performance in the classification task of different data sets (such as MNIST, SVHN, CIFAR10/100, and ILSVRC2012). This method improved the generalization ability of the neural network by optimizing the feature boundary, and at the same time, it had strong robustness in the selection of hyperparameters. In [67], the family of nonlinearities for the neural network were referred to as ‘’Leaky-ReLU.’’ SVM models developed from the study of Leaky-ReLU-type deep neural networks were introduced to transfer classification tasks into linear classification in a particular tensor space. At the end, a generalization bound was developed for deep neural networks. The main idea is to parameterize the neural network, instead of traditional network optimization through weights.

Very simple equipment could still be powerful, by combining straight-forward image analysis along the lines presented, by wing-beat frequency determination. Then a photodiode would be used for the dual purpose of triggering the camera exposure, and for capturing the fundamental wing-beat frequency and the contents of over-tones, as evaluated by Fourier transformation. The frequency spectrum is related to the orientation of the insect [44], which could be inferred also from imperfect images. Basically, a quite powerful but still very cost-effective system could be achieved, by combining the wing-beat frequency analysis described in our earlier insect trap work [68], with the simple imaging approach presented in this paper. We believe that such low-cost systems could be distributed and connected to a central processing unit for achieving very valuable information related to disease vectors, pollinators, as well as agricultural pests in an extended area.

## Figures and Tables

**Table 1:** Performance of different algorithms.

Algorithm Type	Template Matching	Covering	Padding	Detection Rate
Single template matching algorithm	√	×	×	64%
Multi-target template matching algorithm	√	√	×	84%
Our proposed algorithm	√	√	√	92%

**Table 2:** Performance of different methods.

Methods	*TP*	*FN*	*FP*	*Recall*	*Precision*	*F-Measure*
No Classification	86	14	13	86.0%	86.8%	86.4%
Classification	93	6	7	93.9%	93.0%	93.5%

## Data Availability

Data are available from the authors on request.
